# Effect of Hypoxia Preconditioned Adipose-Derived Mesenchymal Stem Cell Conditioned Medium on Cerulein-Induced Acute Pancreatitis in Mice

**DOI:** 10.34172/apb.2020.036

**Published:** 2020-02-18

**Authors:** Kamal Abdolmohammadi, Tayebeh Mahmoudi, Shahrzad Nojehdehi, Lobat Tayebi, Seyed Mahmoud Hashemi, Farshid Noorbakhsh, Alireza Abdollahi, Masoud Soleimani, Behrouz Nikbin, Mohammad Hossein Nicknam

**Affiliations:** ^1^Department of Immunology, School of Medicine, Tehran University of Medical Sciences, Tehran, Iran.; ^2^Student Research Committee, Kurdistan University of Medical Sciences, Sanandaj, Iran.; ^3^Stem Cell Technology Research Center, Tehran, Iran.; ^4^Marquette University School of Dentistry, Milwaukee, WI, 53233, USA.; ^5^Department of Tissue Engineering and Applied Cell Sciences, School of Advanced Technologies in Medicine, Shahid Beheshti University of Medical Sciences, Tehran, Iran.; ^6^Urogenital Stem Cell Research Center, Shahid Beheshti University of Medical Sciences, Tehran, Iran.; ^7^Department of Immunology, School of Medicine, Shahid Beheshti University of Medical Sciences, Tehran, Iran.; ^8^Department of Pathology, School of Medicine, Imam Hospital Complex, Tehran University of Medical Sciences, Tehran, Iran.; ^9^Breast Disease Research Center (BDRC), Tehran University of Medical Sciences, Tehran, Iran.; ^10^Department of Hematology, Faculty of Medical Sciences, Tarbiat Modares University, Tehran, Iran.; ^11^Molecular Immunology Research Center, Tehran University of Medical Sciences, Tehran, Iran.

**Keywords:** Inflammation, Acute Pancreatitis, Cerulein, Mesenchymal Stem Cell, Conditioned Medium, Preconditioning

## Abstract

***Purpose:*** Acute pancreatitis (AP) is an inflammatory disorder distinguished by tissue injury and inflammation of the pancreas. Using paracrine potential of mesenchymal stem cells (MSCs) provides a useful clinical approach in treating inflammatory diseases. We investigated the therapeutic effects of adipose-derived MSC conditioned medium (CM) and hypoxia preconditioned adipose-derived MSC conditioned medium (HCM) in cerulein-induced AP in mice.

***Methods:*** AP was induced in C57BL/6 mice by intraperitoneal injection of cerulein (75 μg/ kg/h × 7 times). One hour following the last injection of cerulein, mice were treated with intraperitoneal injection of CM and HCM (500 µL/mice/30 min × 3 times). Twelve hours following the treatment, serum levels of amylase and lipase were measured. In addition, pancreas pathological changes, immunohistochemical examinations for evaluation of IL-6 expression and pancreatic myeloperoxidase (MPO) enzyme activity were analyzed.

***Results:*** The *in vitro* results of the morphological, differentiation and immunophenotyping analyses confirmed that hypoxia preconditioned MSCs (HP-MSCs) conserve MSCs characteristics after preconditioning. However, HP-MSCs significantly expressed high mRNA level of hypoxia inducible factor 1-α and higher level of total protein. The *in vivo* findings of the current study showed that CM and HCM significantly reduced the amylase & lipase activity, the severity of pancreas tissue injury and the expression of IL-6 and MPO enzyme activity compared with the AP group. However, no significant difference between CM and HCM groups was demonstrated.

***Conclusion:*** Use of CM and HCM can attenuate cerulein-induced AP and decrease inflammation in the pancreas tissue in AP mice.

## Introduction

Acute pancreatitis (AP) is a gastrointestinal inflammatory disease characterized by acinar cells injury and inflammation in pancreatic tissue. AP is the most common cause of hospital stays among gastrointestinal diseases.^1,2^ Clinical manifestation of AP ranges from mild (80%-85%) to severe forms (15%-20%). Gallstone and alcohol abuse are two major causes for AP.^1-3^

Previous studies have demonstrated acinar cell death, edema and histopathological changes induced by the activation and release of pancreatic enzymes in early phases of pancreatitis.^4-6^ Along with pancreatic enzyme activation (trypsin, amylase, lipase), activation of NF-KB also occurs in acinar cells.^5^ Moreover, inflammatory mediators—including tumor necrosis factor alpha (TNF-α), interleukin 1 beta (IL-1β), IL-6, IL-33, chemokines and neutrophilic myeloperoxidase (MPO), which are produced by immune cells and injured acinar cells—can exacerbate the inflammatory cascade in the AP patients.^3,5,7-13^

Currently, endoscopic retrograde cholangiopancreatography (ERCP), non-steroidal anti-inflammatory drugs (NSAIDs), use of prophylactic antibiotics and enteral nutrition are the available treatment options for AP. Nevertheless, the mortality rate of the severe stage of AP is around 30%.^2,9^ These strategies chiefly target the symptoms rather than the cause of the disease; therefore, the development of new treatment approaches, such as cell-based therapy, is required for more efficient management of this complicated gastrointestinal disease.^2,9,14,15^

Mesenchymal stem cells (MSCs) and their secreted molecules—which possess regenerative, anti-inflammatory and antioxidative properties—have been suggested as a potential therapeutic approach in many inflammatory and immune-mediated disorders.^16-22^

Jung et al, for the first time in 2011, reported the protective effects of MSCs in treating AP.^23^ Subsequently, several studies demonstrated that MSC therapy can decrease inflammatory mediators and mitigate histopathological changes in pancreatitis through direct differentiation to acinar cells or indirect immunomodulatory effects. Nevertheless, clinical trial studies have not yet been performed to evaluate the effects of MSC therapy in AP.^24-26^

The strong paracrine activity of MSCs is a usable capability to treat many autoimmune and inflammatory disorders.^20,27,28^ Recent studies reported the protective effects of MSC-derived conditioned medium (MSC-CM) or MSC-derived extracellular vesicles (MSC-EVs) in some animal models of diseases.^17,20,22,27,29,30^ Proteomics analysis of MSC-CM has identified more than 100 proteins (including cytokines, chemokines and growth factors) with anti-inflammatory, anti-apoptotic, anti-fibrotic and regenerative effects.^28,31^

It has been shown that exposure of MSCs to hypoxic conditions might enhance their immunomodulatory and regenerative properties via over-expression of cytoprotective genes and secretory factors.^28,32^ Hypoxia inducible factor 1-α (HIF-1α) has a crucial role in regard to the upregulation of these genes and factors.^33,34^ Accordingly, hypoxia preconditioned MSCs can attenuate inflammation, tissue injury and fibrosis in some of the experimental animal models.^34,35^

To our knowledge, the protective effects of adipose-derived MSC (AD-MSC) conditioned medium (CM) and hypoxia preconditioned adipose-derived MSC conditioned medium (HCM) have not yet been investigated in the therapy of AP. Therefore, this study investigates the therapeutic effects of CM and HCM, which are assessed in mice with cerulein-induced AP.

## Materials and Methods

### Animals

Male C57BL/6 mice (6-8 weeks, 18-25 g) were obtained from the Pasteur Institute of Tehran, Iran. The mice were kept under standardized animal housing conditions.

### Isolation, culture and characterization of AD-MSCs

Epididymal adipose tissue of C57BL/6 mice was removed and homogenized using 0.1% collagenase type I (Gibco, UK). Isolation and culture of AD-MSCs were performed as previously described.^29^ Briefly, the adipose tissue was homogenized in DMEM/F12 (Gibco, UK) and centrifuged at 1500 rpm for 15 minutes. The pellet was suspended in DMEM/F12 with 10% FBS (Gibco, UK), 100 U/mL of penicillin and 100 µg/mL of streptomycin. AD-MSCs were incubated in the standard cell culture conditions.

After obtaining of 70%-80% confluency, MSCs were trypsinized using trypsin 0.05% (Sigma, USA) and 0.02% EDTA, then subcultured. Characterization of MSCs was performed by morphological evaluation, differentiation assay and flow cytometry analysis at the second passage.

### Characterization of AD-MSCs with differentiation assay

Differentiation potency of AD-MSCs into osteocyte and adipocyte lineage was assessed by culturing in osteogenic differentiation media (containing glycerol phosphate (10 mM), dexamethasone (100 mM) and ascorbic acid‐2 phosphate (5 g/mL)) and adipogenic differentiation media (containing indomethacin (100 mM), 3‐isobutyl‐methylxanthine (0.5 mM), dexamethasone (250 mM) and insulin (5 mM)) for 3 weeks. Finally, mineralization of osteocytes was identified by staining with Alizarin Red S, and entity of oil vacuoles in the adipocytes was evaluated by Oil Red O staining. Briefly, media was discarded and MSCs were rinsed by phosphate-buffered saline (PBS). Paraformaldehyde (4%) was used for cell fixation (20 min/4ºC); then after washing with PBS, MSCs were stained with 2% Alizarin Red S and 0.5% Oil Red O solution, respectively (10 min/RT).^17,29^

### Characterization of AD-MSCs with flow cytometry analysis

Surface markers for MSCs characterization were analyzed using monoclonal anti-mouse antibodies, including anti‐CD44-FITC (561859 BD Biosciences), anti‐CD105-PerCP Cy5.5 (120415 BioLegend), anti‐CD34-PE (551389 BD Biosciences) and anti‐CD45-FITC (553079 BD Biosciences), along with isotype control antibodies. Preparation of samples for immunophenotyping analysis was performed as previously described.^29^ The samples were assessed using BD FACS Calibur flow cytometer (BD biosciences, San Jose, CA, USA) and analyzed by FlowJo 7.6 software.

### Hypoxic preconditioning and characterization of hypoxia preconditioned-MSCs (HP-MSCs)

AD-MSCs with 70%-80% confluency at second passage were cultured under hypoxic conditions (2-5% oxygen concentration, 5% CO_2_ concentration and balanced N_2_) for 48 hours. HP-MSCs were characterized by morphological evaluation, differentiation assay and flow cytometry analysis as previously described.

### Identification of HP-MSCs by real-time PCR

To identify the effects of hypoxic preconditioning, mRNA expression level of HIF-1α was analyzed using quantitative real-time PCR. Total RNA was extracted from the AD-MSCs under standard and hypoxic cell culture conditions by RNA X plus (Sina Clone Co., Tehran, Iran). Next, cDNA synthesis was performed using the random hexamer primer, dNTP and M-MLV reverse transcriptase enzyme (Yekta Tajhiz Co., Tehran, Iran). The real-time PCR was completed with the Applied Biosystems StepOnePlus™ system, using SyberGreen Master Mix (Sina Clone Co., Tehran, Iran) and mouse HIF-1α specific primers. Real-time PCR was conducted with the following conditions: initial denaturation stage (95°C/2 min), cycling stage (95°C/5 s and 60°C for 30 seconds, 40 cycles) and melt curve stage (95°C/15 s, 60°C for 1 minute and 95°C/15 s). The mitochondrial ribosomal protein S16 (MRPS16) mRNA expression level of a reference mouse was used for normalization. The relative gene expression was calculated by the ΔΔCt method. Mouse gene-specific primers sequences are listed in Table 1.

**Table 1:** Mouse gene-specific primers sequences

**Gene name**	**Primer sequence (5’………3’)**	**Accession number**
HIF-1α	Forward: TTGGCAGCGATGACACA Reverse: CGATGAAGGTAAAGGAGACATT	NM_001313919.1
MRPS16	Forward: TCGGACGCAAGAAAACAG Reverse: CCACCACCCTTCACACG	NM_013647.2

### Preparation and total protein assay of CM **&** HCM

Conditioned medium preparation protocol has been reported in several previous studies.^17,22,36^ Briefly, AD-MSCs at second passage with 70%-80% confluency were incubated in serum-free DMEM/F12 media under standard and hypoxic conditions. After 48 hours, the CM of AD-MSCs and HP-MSCs was collected. CM and HCM were centrifuged (1500 rpm for 5 minutes), filtered (0.22 µm filter), stored (-80°C) and utilized for injection into experimental groups in the treatment procedures.

Before the treatment procedures, the total protein concentration of CM and HCM was measured using the Pierce™ BCA protein assay kit (Thermo Fisher Scientific, Waltham, MA). Optical density (OD) measurements were performed at 570 nm, and the protein concentration of CM and HCM was calculated by standard curve obtained from serial dilutions of bovine serum albumin (BSA). The results were reported in µg/mL.

### Induction of disease, experimental grouping and treatment procedure

Male C57BL/6 mice were induced with AP at 6-8 weeks-old by intraperitoneal (i.p.) injection of cerulein, as previously described.^9^ Briefly, mice received seven injections of 75 μg/kg cerulein (C9026 Sigma-Aldrich) dissolved in 200 µL sterile saline at hourly intervals intraperitoneally. Forty-two mice were divided into six experimental groups (n = 7 mice/group). The normal group (Normal) received sterile normal saline (200 µL/h × 7 times, i.p.). The AP group received cerulein injections (75 µg/kg/h, dissolved in 200 µL sterile normal saline, i.p.). One hour after the last cerulein injection, Normal and AP experimental groups received injections of 1.5 mL sterile PBS (500 µL/mice/30 min × 3 times, i.p.).

**Figure 1:**
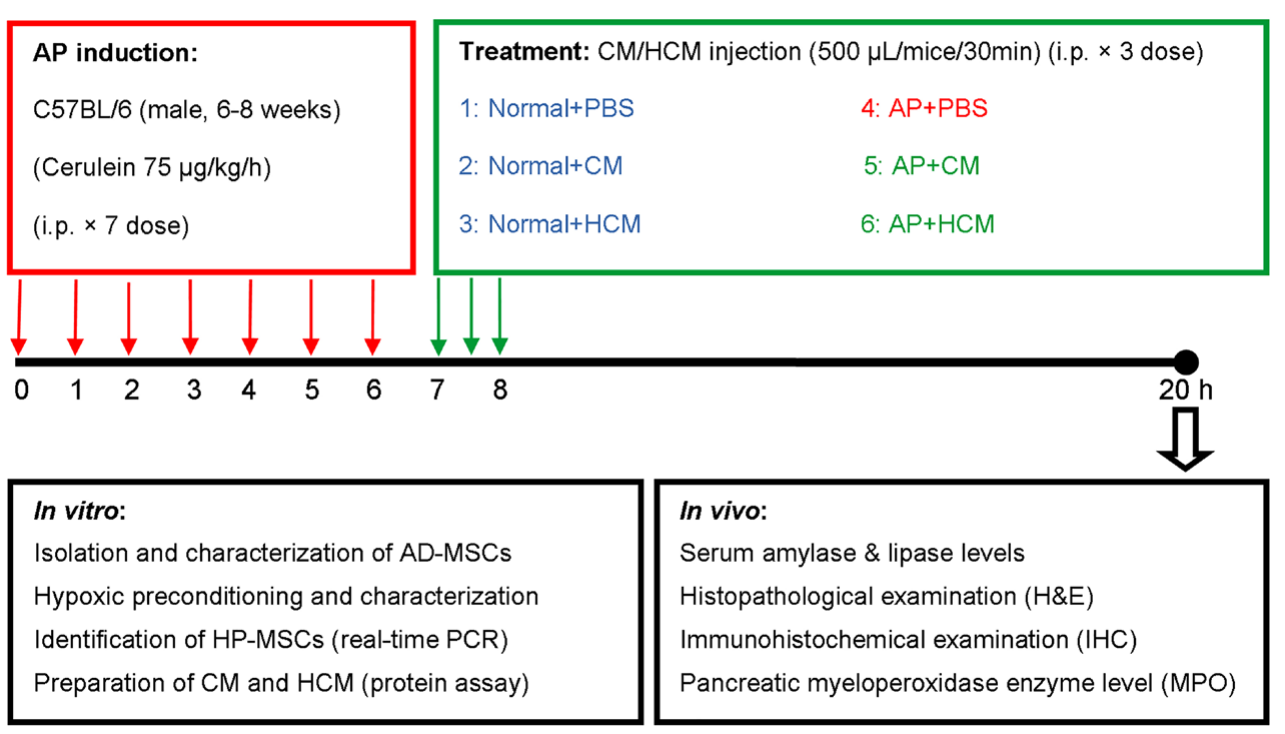
Study design for AP induction, treatment procedure, *in vitro* and *in vivo* experiments. Abbreviations: AD-MSCs: adipose-derived mesenchymal stem cells, AP: acute pancreatitis, CM: adipose-derived MSC conditioned medium, HCM: hypoxia preconditioned adipose-derived MSC conditioned medium, HP-MSCs: hypoxia preconditioned mesenchymal stem cells, i.p.: intraperitoneal.

For the treatment procedure, (AP+CM) and (Normal+CM) experimental groups were treated with injection of 1.5 mL CM (500 µL/mice/30 min × 3 times, i.p.). Two other groups of mice—(AP+HCM) and (Normal+HCM)—were treated with HCM. Twelve hours after treatment, all mice in the experimental groups were euthanized. Under the sterile conditions, pancreatic tissues were quickly removed for evaluation of MPO enzyme activity, along with histopathological and immunohistochemical examinations. Whole blood samples were obtained to evaluate levels of amylase and lipase (Figure 1).

### Determination of levels of serum amylase and lipase

Determination of amylase and lipase, along with histopathological examinations, were used for characterization of AP induction in experimental animal models and evaluation of treatment effects. The blood samples were centrifuged at 2500 rpm for 10 minutes at 4°C. Serum samples were stored at -80°C in an ultra-low temperature freezer (New Brunswick, Eppendorf Co.). A biochemistry autoanalyzer (Roche Hitachi 917) measured serum levels of amylase and lipase using the calorimetric method (Pars Azmoon Co., Tehran, Iran).

### Histopathological examination

At the end of the experimental procedure, a part of the pancreatic tissue was used for the histopathological examination. After washing with PBS, pancreatic samples were fixed in 10% buffered formaldehyde following the histopathological processing,^9,37^ and stained with hematoxylin and eosin (H&E) to investigate acinar cell vacuolization, leukocyte infiltration, acinar cell necrosis, edema, and hemorrhage. Finally, the pancreatic tissue sections were examined using the optical microscope at 100X and 400X. Six sections per tissue and seven animals per group were analyzed.

### Immunohistochemical examination

The pancreatic sections collected from the experimental groups were immunostained with IL-6, which is an important inflammatory biomarker in AP. Briefly, after deparaffinization (65-70ºC for 30 minutes) and hydration, antigen retrieval was performed using Universal HIER Ag Retrieval Reagent (Abcam Biotechnology Co., ab208572) (95°C for 20-60 minutes). The samples were blocked using blocking buffer (goat serum, BSA, Tween 20, Triton X100, PBS) (37°C for 60 minutes), then incubated with rabbit polyclonal antibody of anti-mouse IL-6 (Abcam Biotechnology Co., ab208113) (1:200 in PBS+1% BSA) overnight at 4°C. Endogenous peroxidase of tissue samples was quenched with 0.3% hydrogen peroxide (15-20 minutes at dark room). Following washing with PBS, sections were detected with a sheep anti-rabbit Ig secondary antibody conjugated to HRP (Padza Co., Tehran, Iran, pz5684) (1:500 in PBS+1% BSA), then a solution of 0.1% 3, 3-diaminobenzidine and 0.02% H_2_O_2_. Finally, the samples were stained with hematoxylin and prepared after dehydration and mounting. The samples were examined using the optical microscope at 100X and 400X. Six sections per tissue and seven animals per group were analyzed by ImageJ Software.

### Evaluation of myeloperoxidase enzyme activity

Pancreas MPO enzyme level was measured by the MPO assay kit (Nampox^TM^, Navand Salamat Co., Uremia, Iran), according to the standard protocol. Briefly, frozen pancreatic tissue samples were weighed and homogenized in sample buffer 1X (pH=6.0) containing 0.5% hexadecyl trimethyl ammonium bromide. The samples were centrifuged at 10 000 rpm for 10 minutes in 4°C. Absorbance measurements were recorded at 450 nm in the supernatant using TMB and H_2_O_2_. The results of MPO assay were reported as the OD/mg tissue.^38^

### Statistical analysis

Data was analyzed using GraphPad Prism 5 software, and experimental groups were compared using one-way ANOVA with Tukey’s multiple comparison or Student’s *t* test statistical analysis. Data was presented as a mean ± standard deviation (SD). A *P* value < 0.05 was set as the statistical significance level.

## Results and Discussion

### AD-MSCs and HP-MSCs characterization by morphological evaluation, differentiation assay and flow cytometry analysis

**Figure 2:**
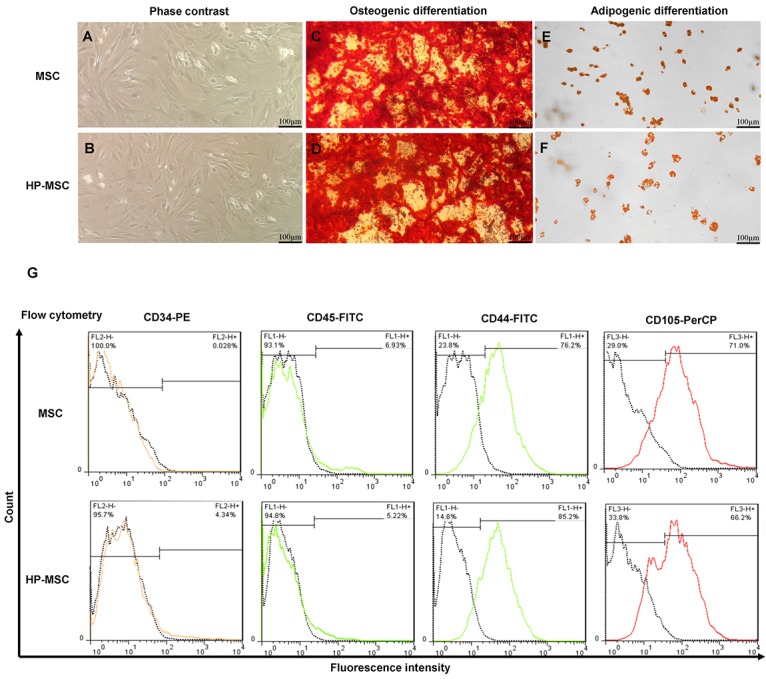
HP-MSCs preserve mesenchymal stem cell characteristics after hypoxic preconditioning (2-5% O_2_ for 48 h). Representative images of MSCs (**a**) and HP-MSCs (**b**) by a phase-contrast microscope. Osteogenic (**c**, **d**) and adipogenic (**e**, **f**) differentiation potential of MSCs and HP-MSCs after staining by Alizarin Red S and Oil Red O, respectively. The expression level of mesenchymal stem cell surface markers on MSCs and HP-MSCs was analyzed by flow cytometry analysis (**g**). All experiments were performed at the second passage. Abbreviations: HP-MSC: hypoxia preconditioned mesenchymal stem cell, MSC: adipose-derived mesenchymal stem cell.

Morphological evaluation of MSCs and HP-MSCs showed typical spindle-shaped fibroblast-like adherent cells after *in vitro* expansion using the inverted phase-contrast microscopy (Figure 2A, B).

Following 3 weeks of culture in the differentiation media, the osteogenic and adipogenic potency of MSCs and HP-MSCs were confirmed using Alizarin Red S and Oil Red O, respectively. The results of differentiation assay revealed that MSCs and HP-MSCs did not vary in their differentiation into osteoblasts and adipocytes (Figure 2C-F).

According to the recommended criteria for the definition of MSCs,^39^ immunophenotyping analysis of MSCs and HP-MSCs showed that both MSCs and HP-MSCs expressed low levels of hematopoietic surface markers—like CD34 and CD45—while these cells expressed increased levels of mesenchymal surface markers—including CD44 and CD105. The percentage of each surface marker in MSCs and HP-MSCs is presented in Figure 2G. The results of the morphological evaluation, differentiation potency assay and immunophenotyping analysis showed that HP-MSCs preserve MSC characteristics after hypoxic preconditioning (2%-5% O_2_ for 48 hours).

### Hypoxic preconditioning verification by the mRNA expression level of HIF-1α

**Figure 3:**
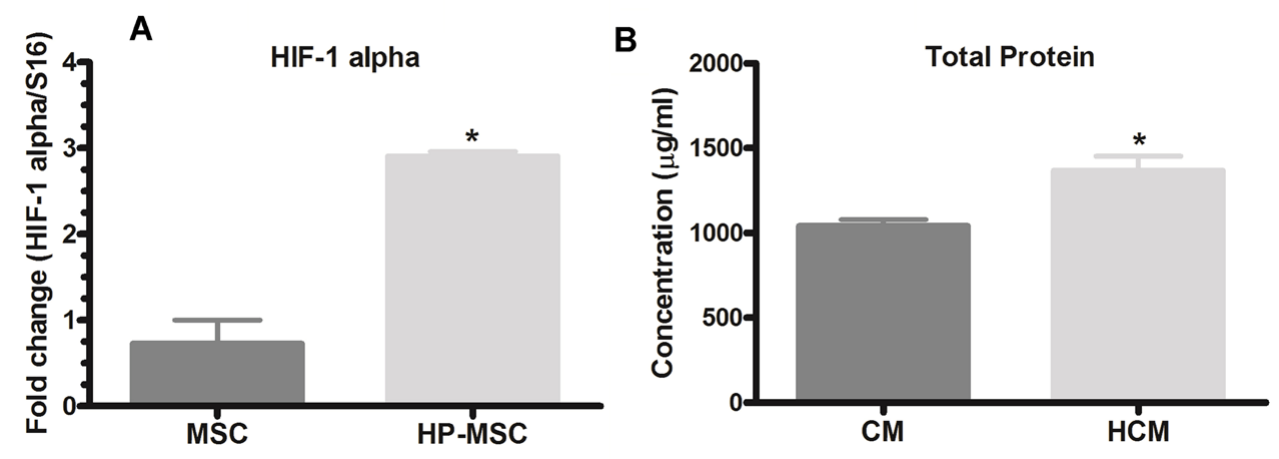
Hypoxic preconditioning (2-5% O_2_ for 48 h) increases the expression of HIF-1α in HP-MSCs and total protein concentration in HCM. Expression level of HIF-1α (**a**) was analyzed by real-time PCR. HP-MSC expresses a high level (3.98-fold) of HIF-1α in comparison to the MSC (^*^P < 0.05). Total protein concentration (**b**) was measured by BCA protein assay kit. HP-MSCs produces higher levels (1.31-fold) of proteins compared to MSCs cultured in standard conditions (^*^P < 0.05). All experiments were analyzed in three series. Abbreviations: CM: adipose-derived MSC conditioned medium, HCM: hypoxia preconditioned adipose-derived MSC conditioned medium, HIF-1α: hypoxia inducible factor 1-α, HP-MSCs: hypoxia preconditioned mesenchymal stem cells.

To verify the effect of hypoxia on MSCs, the mRNA expression level of HIF-1α was analyzed in AD-MSCs and HP-MSCs by real-time PCR. Results showed that MSCs cultured in hypoxic conditions (HP-MSC) significantly expressed a high level of HIF-1α in comparison to the MSCs cultured in standard conditions (MSC) (3.98-fold, *P* < 0.05) (Figure 3A). To obtain these results, three series of cultured MSCs and HP-MSCs were extracted and analyzed.

### Total protein assay of CM and HCM

After 48 hours incubation of MSCs under standard and hypoxic conditions in serum-free DMEM/F12 media, protein concentration in MSC and HP-MSC derived CM were calculated using the Pierce™ BCA protein assay kit. The results demonstrated that MSCs cultured for 48 h in hypoxic conditions (HP-MSC) produced significantly higher (*P* < 0.05) levels of proteins compared to MSCs cultured in standard conditions (MSC) (Figure 3B).

*In vitro* pre-conditioning can improve the effects of MSC and CM via over-expression of cytoprotective genes.^34,40,41^ Some notable studies demonstrated that hypoxic preconditioning increases the expression level of HIF-1α, which acts an important gene in the upregulation of cytoprotective factors. Previous studies reported that hypoxic conditions modulate the paracrine activity, upregulates the secretable factors and increases the protein levels of HP-MSCs similar to that of MSCs cultured in the normal conditions.^32,42^

Our *in vitro* findings demonstrate that hypoxic preconditioning of adipose-derived MSCs (2-5% O_2_ for 48 hours) does not change the morphology, differentiation potency or phenotyping characteristics of MSCs (Figure 2), yet it significantly increases the expression of HIF-1α in HP-MSCs and total protein concentration in CM derived from HP-MSCs (Figure 3).

### Effect of CM and HCM on serum amylase and lipase levels

**Figure 4:**
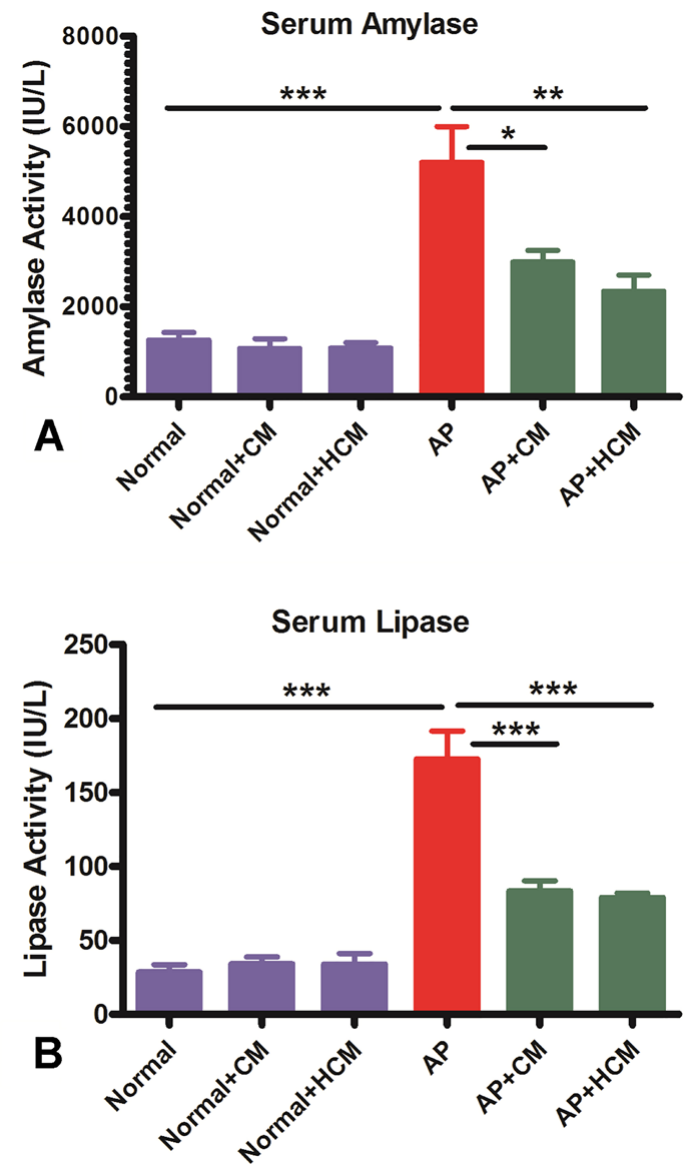
CM and HCM injections decrease serum amylase and, especially, lipase levels in AP mice. A biochemistry autoanalyzer was used for measuring serum levels of amylase (**a**) and lipase (**b**). Data is expressed as mean ± SEM (n=7). One-way ANOVA test was used for comparing differences between experimental groups. *P < 0.05, **P < 0.01, ***P < 0.001. Abbreviations: AP: acute pancreatitis, CM: adipose-derived MSC conditioned medium, HCM: hypoxia preconditioned adipose-derived MSC conditioned medium.

One of the criteria for diagnosing AP is elevated levels of amylase and lipase.^2,13,43^ The cerulein-induced AP mice (AP group) is characterized by a significantly higher level of serum amylase and lipase (*P* < 0.001, Figure 4A, B). As demonstrated in Figure 4A and 4B, intraperitoneal injection of CM and HCM significantly reduced levels of amylase and lipase activity compared with the AP group (*P* < 0.05, *P* < 0.01, *P* < 0.001). There was no statistically significant difference in levels of amylase and lipase between the Normal mice compared to the Normal+CM and Normal+HCM mice.

Despite the progression in understanding the immunopathological mechanisms of AP, there is still no satisfactory approach for curing this inflammatory disease, as current approaches for the treatment of AP chiefly target the symptoms rather than relieving pancreatic tissue injury.^1,2,5,6,9,11,13,44,45^ Therefore, an efficient therapeutic approach with an anti-inflammatory and regenerative potential is useful for the treatment of AP patients. In this research, we aimed to explore the therapeutic effect of CM and HCM of AD-MSCs in an experimental model of AP.

Previous studies recommend that the use of MSCs can mitigate experimental AP, inhibit inflammation and ameliorate pancreas tissue damage.^23-25^ MSC-based therapy utilizing all the MSC components faces some obstacles, such as survival rate of transplanted cells and ethical concerns.^22,29,46^ Regarding the limitations of MSC use in the treatment of AP, there is no warranted clinical trial in the therapy of AP.^24,25^ Some studies reported the anti-inflammatory and regenerative effects of MSC-derived CM.^17,22,36^ Proteomics analysis of MSC-CM showed several cytokines, chemokines and growth factors with anti-inflammatory, anti-apoptotic, anti-fibrotic and regenerative properties.^28,31^ Therefore, it seems that CM, as a multi-functional drug, can be a suitable and simplified new approach for the treatment of AP. Similar to other studies that reported the anti-inflammatory and regenerative effects of MSC in animal models of AP,^24,25^ our *in vivo* results show that CM and HCM can significantly decrease the serum amylase and, especially, lipase levels (Figure 4A, B).

### Effect of CM and HCM on histopathological findings

**Figure 5:**
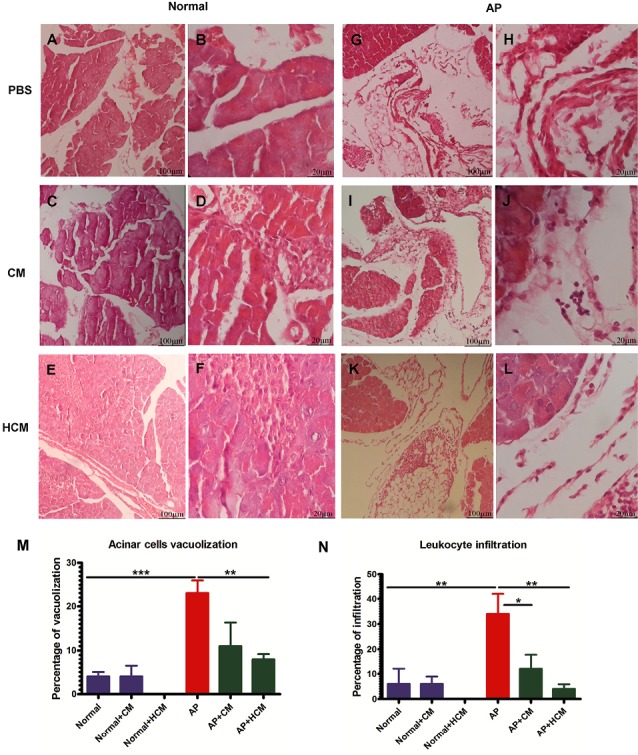
CM and HCM injections attenuate acinar cell vacuolization and decrease the leukocyte infiltration in the pancreatic tissue of AP mice (**i-l**). Cerulein significantly leads to tissue injury and leukocyte infiltration in pancreatic tissue of AP mice (**g, h**) compared to the Normal group (**a, b**). Hematoxylin and eosin (H&E) staining was used for histopathological evaluations using the optical microscope at 100X and 400X. The results of microscopic examination are reported as a percentage of acinar cell vacuolization (**m**), leukocyte infiltration (**n**), acinar cell necrosis, edema and hemorrhage (**Figures S1-S3**). One-way ANOVA test was used for comparing differences between experimental groups. *P < 0.05, **P < 0.01, ***P < 0.001. Abbreviations: AP: acute pancreatitis, CM: adipose-derived MSC conditioned medium, HCM: hypoxia preconditioned adipose-derived MSC conditioned medium.

Acinar cell injury and inflammatory cell accumulation are the essential indicators to determine the severity of tissue damage in AP.^3,9^ Histopathological results showed that cerulein significantly lead to tissue injury and leukocyte infiltration in pancreatic tissue of AP mice (Figure 5G, H) compared to the Normal group (Figure 5A, B). After treatment of AP mice with CM (Figure 5I, J) and HCM (Figure 5K, L), leukocyte infiltration and acinar cell vacuolization were significantly reduced in the treated groups as compared to the AP group (Figure 5M, N). However, there was no significant difference in the acinar cell necrosis, edema and hemorrhage between experimental groups (Supplementary file 1, Figures S1-S3).

Histopathological findings of the current study demonstrated that CM and HCM can attenuate acinar cell vacuolization and decrease the leukocyte infiltration in cerulein-induced AP (Figure 5). Because of the short time period between treatment intervention and histopathological examination, it seems the anti-inflammatory effects of the CM and HCM are more obvious than the regenerative effects. MSC-CM includes many anti-inflammatory mediators—mainly PGE2, IDO, HGF, TGF-β and IL-10—which can inhibit the inflammatory cascade and tissue injury in several inflammatory diseases.^17,22,36,47^

### Effect of CM and HCM on immunohistochemical findings

**Figure 6:**
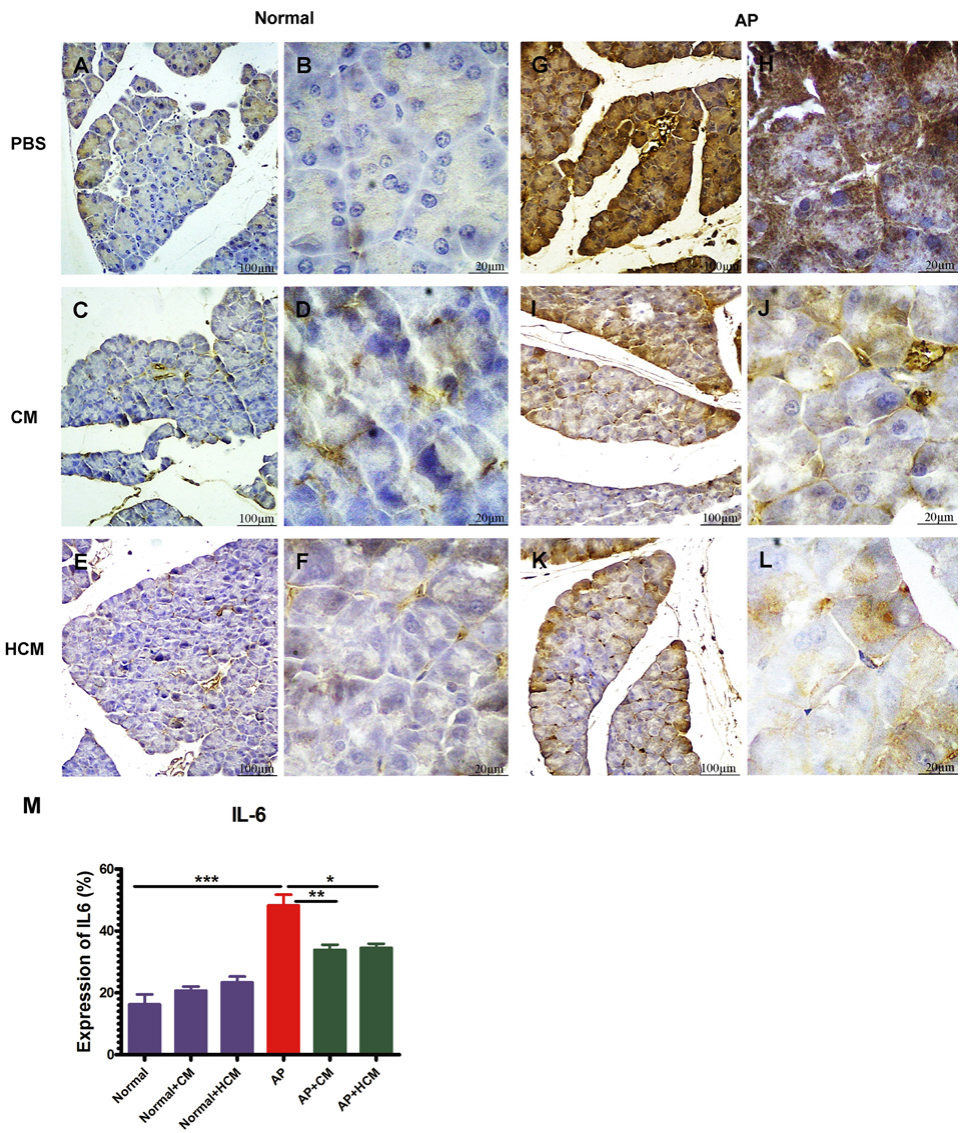
CM and HCM injections reduce the expression of IL-6 in the parenchyma areas in the pancreatic tissues of AP mice (**i**-**l**) compared to the AP group (**g**, **h**). CM and HCM injection to the Normal mice does not significantly affect the increase or decrease of IL-6 expression (**a**-**f**). Immunohistochemical (IHC) staining was used for IL-6 expression examination after the treatment. The samples were examined using the optical microscope at 100X and 400X. The results of six sections per tissue and seven animals per group were analyzed by Image J Software (**m**). One-way ANOVA test was used for comparing differences between experimental groups. *P < 0.05, **P < 0.01, ***P < 0.001. Abbreviations: AP: acute pancreatitis, CM: adipose-derived MSC conditioned medium, HCM: hypoxia preconditioned adipose-derived MSC conditioned medium.

Immunohistochemical staining results showed a high expression of IL-6 in the parenchyma areas of pancreatic tissues in AP mice (Figure 6G, H) compared to the Normal group (Figure 6A, B). Treatment with CM (Figure 6I, J) and HCM (Figure 6K, L) significantly decreased the expression of this pro-inflammatory cytokine compared to the AP group (Figure 6M). CM and HCM injection to the Normal mice did not significantly affect the increase or decrease of IL-6 expression (Figure 6C-F).

**Figure 7:**
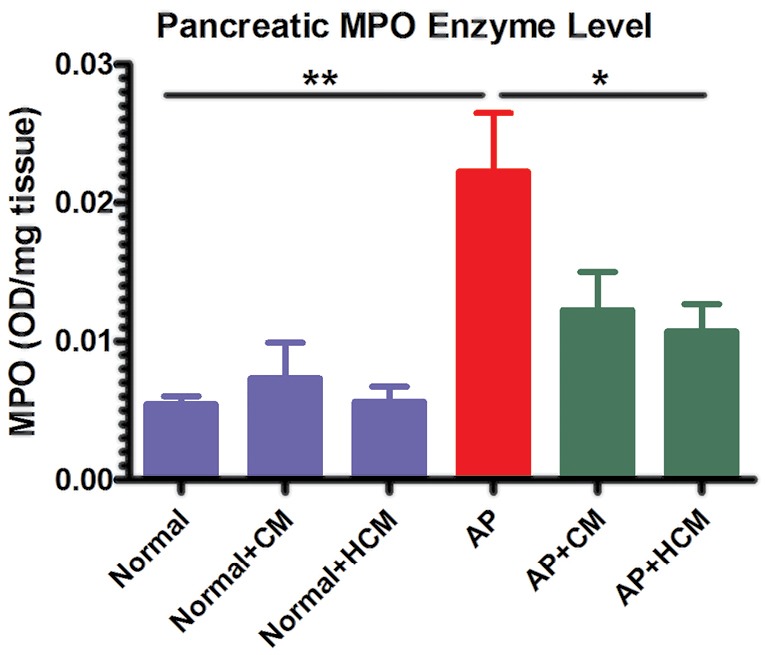
Mice treated with HCM demonstrate significantly reduced levels of MPO enzyme compared to the AP group. However, there is no significant difference in the MPO levels after treatment with CM. MPO enzyme activity was measured by the MPO assay kit and OD measurements were recorded at 450 nm. The results are reported as the OD/mg tissue. Data is expressed as mean ± SEM (n=7). One-way ANOVA test was used for comparing differences between experimental groups. *P < 0.05, **P < 0.01. Abbreviations: AP: acute pancreatitis, CM: adipose-derived MSC conditioned medium, HCM: hypoxia preconditioned adipose-derived MSC conditioned medium, MPO: myeloperoxidase, OD: optical density.

Numerous animal models of AP have been generated to study pathogenic mechanisms and investigate therapeutic approaches in AP. Experimental AP has been created by cerulein, sodium taurocholate (Na TCA), L-arginine, choline-deficient diet and autoimmune pancreatitis models. Cerulein-induced experimental AP is the most frequently used AP model, and is similar to human disease.^23,24^ In the present study, we utilize the cerulein-induced model to evaluate the ability of CM and HCM in the treatment of AP. Cerulein induces high levels of reactive oxygen species and cytokines production, such as IL-6 in acinar cells by NF-KB stimulation.^44^ Previous studies showed that there is a positive correlation between levels of IL-6 and severity of AP.^48,49^ In our AP model, immunohistochemical results demonstrate significant reduction in expression of IL-6 after treatment with CM and HCM (Figure 6). In agreement with the current study, Tu et al demonstrated that MSCs with anti-inflammatory effects can mitigate severe AP in rats via decreasing the expression of inflammatory cytokines, like IL-6.^50^

### Effect of CM and HCM on MPO enzyme activity

Cerulein regulates infiltration, while inflammatory cell activation mainly affects innate immune cells, such as neutrophils within the pancreatic tissue.^51^ MPO is one of the principal enzymes in the azurophilic granules of leukocytes, especially neutrophils. Therefore, evaluation of MPO enzyme level shows the presence and activity of neutrophils within the inflamed tissue.^8^ As shown in Figure 7, the levels of MPO enzyme were significantly increased in the pancreas of AP mice compared to the Normal mice (*P* < 0.01). Mice that were treated with HCM demonstrate significantly reduced levels of MPO enzyme compared to the AP group (*P* < 0.05). But, there was no significant difference in the MPO levels after treatment with CM.

Several studies demonstrated that cerulein regulates infiltration and activation of inflammatory cells—mainly innate immune cells—within the pancreatic tissue via upregulation of intercellular adhesion molecule-1 in pancreatic acinar cells surface.^51^ Neutrophils and MPO within their azurophilic granules play an important role in the immunopathogenesis of AP.^4,7,8,14,15^ Our investigation presents for the first time that intraperitoneal injection of HCM, unlike CM, can decrease the MPO enzyme level in AP mice (Figure 7).

Previous *in vivo* studies demonstrated that hypoxic preconditioning of MSCs can promote their anti-inflammatory and regenerative effects in some experimental animal models.^28,32,34,35^ However, our *in vivo* findings revealed that there was no significant difference between the effect of CM and HCM on serum amylase and lipase levels, pathological changes, IL-6 expression or MPO enzyme level in the cerulein-induced AP (Figures 4-7). We used a single dose of CM and HCM in the current study. Some previous studies demonstrated that repeated doses of MSCs-derived CM are more effective compared to the single dose,^52^ so future investigations involving a dose-dependent manner can be helpful for understanding this issue in the AP animal models. In addition to assay total protein concentration, specific protein assay and factor discovery in the HCM can be the next step for evaluating the HCM effects in the immunopathogenesis of AP. Intravenous versus intraperitoneal injection of CM and HCM may also have different effects in the outcome of AP. Finally, use of different animal models of AP will provide additional insights into the effects of CM and HCM in the therapy of AP.

## Conclusion

In brief, we showed that injection of CM of MSCs and HP-MSCs attenuates AP and reduces inflammation in cerulein-induced AP in mice. Therefore, use of CM and HCM can be considered as a cell-free treatment in the future studies in this field.

## Ethical Issues

All of the animal studies were conducted with the approval of the Ethics Committee of the School of Medicine, Tehran University of Medical Sciences, Tehran, Iran (Code of Ethics: IR.TUMS.MEDICINE.REC.1396.3953).

## Conflict of Interest

Authors declare no conflict of interest in this study.

## Acknowledgments

This study was supported by the research grants from the Tehran University of Medical Sciences [grant number 96-03-30-36346]. We appreciate all collaborators in the Stem Cell Technology Research Center, for their technical assistance in this project.

## Supplementary File


Supplementary file 1 contains Figures S1-S3.
