# Supplementary figures and images for: Effect of Hypoxia Preconditioned Adipose-Derived Mesenchymal Stem Cell Conditioned Medium on Cerulein-Induced Acute Pancreatitis in Mice
Source: Adv Pharm Bull. 2020 Feb 18;10(2):297–306. doi: 10.34172/apb.2020.036 (PMC7191232; doi:10.34172/apb.2020.036)

## Supplementary File 1

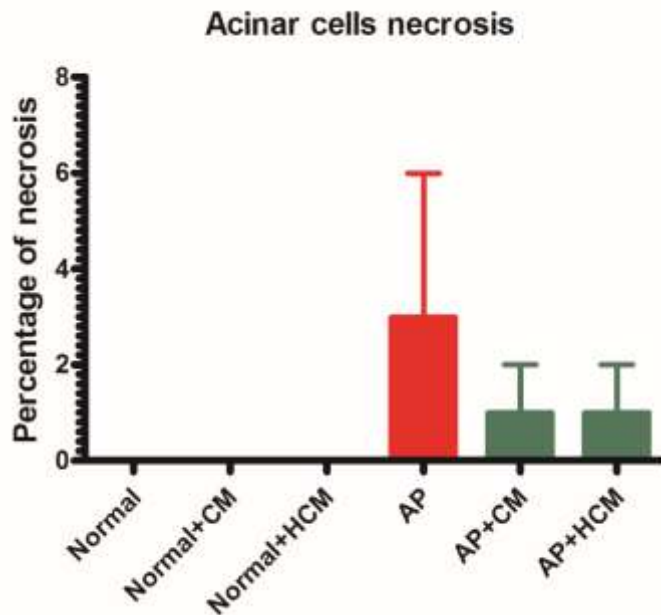

Figure S1. Acinar cells necrosis.

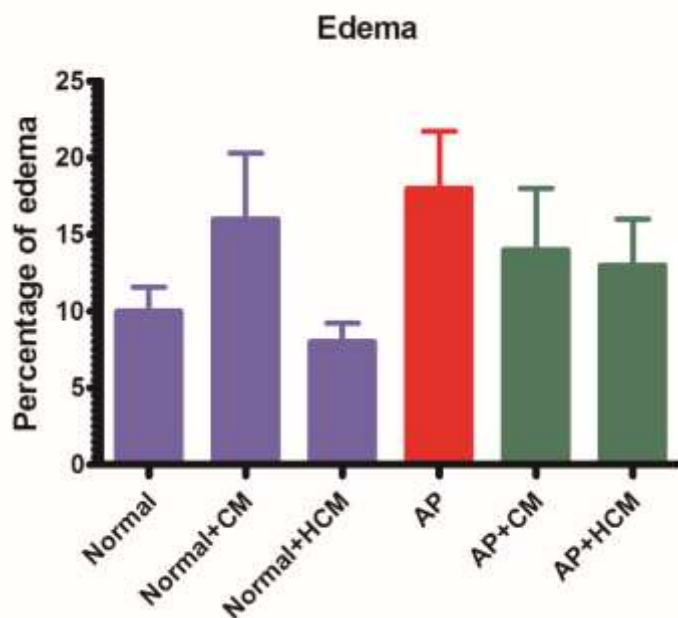

Figure S2. Edema.

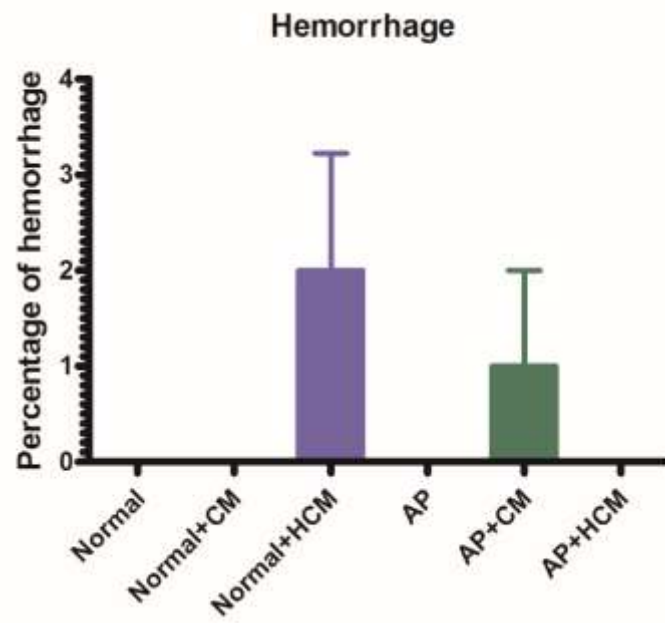

**Figure S3.** Hemorrhage.

Supplement: Supplementary file 1 — contains Figures S1-S3. [file apb-10-297-s001.pdf]
